# A Gamified Mobile Health Intervention to Promote Physical Activity, Executive Function, and Mental Health in College Students: Randomized Controlled Trial

**DOI:** 10.2196/82769

**Published:** 2026-04-07

**Authors:** Ming Yang, Yingjun Guo, Zhen Li, Lei Jiang, Yanan Gao, Sen Yang, Zhixiong Zhou

**Affiliations:** 1 Institute of Artificial Intelligence in Sports, Capital University of Physical Education and Sports, No.11, North Third Ring West Road, Haidian District, Beijing, 100191, China, 86 13552505679; 2 School of Physical Education, Yantai University, Yantai, Shandong, China; 3 Beijing Sport University Press, Beijing Sport University, Beijing, Beijing, China; 4 School of Physical Education and Sport Science, Fujian Normal University, Fuzhou, Fujian, China

**Keywords:** mHealth, eHealth, gamification, physical fitness, psychological well-being, cognitive function, adherence

## Abstract

**Background:**

College students commonly experience suboptimal health conditions, including insufficient physical activity (PA), excessive body weight, and declining physical fitness. Traditional interventions face low adherence, while gamified mobile health (mHealth) programs may improve engagement and outcomes.

**Objective:**

This study aimed to evaluate the feasibility and effectiveness of a novel gamified, incentive-based mHealth intervention on primary outcomes (PA and adherence) and secondary outcomes (physical fitness, body composition, executive function [EF], and mental health).

**Methods:**

A 2-arm parallel-group randomized controlled trial (RCT) was conducted in 2025 at Yantai University with 160 college students (18‐25 years; BMI 18.5‐30.0) who were randomized 1:1 (computer-generated, sex-stratified blocks of 4; concealed allocation) to the intervention group (IG) or control group (CG; n=80 each); major exclusions were contraindications to exercise, severe physical/mental illness, recent PA interventions, or psychotropic medication use. Both used the same fitness watch–app system and identical PA targets (≥150 min moderate-to-vigorous physical activity [MVPA] per week or ≥900 metabolic equivalent-minutes [MET-min] per week); IG additionally received team-based gamification (competition, points/leaderboards, feedback, and rewards), while CG received monitoring only. PA and adherence were monitored throughout the 8-week intervention; other outcomes were assessed at baseline and 8 weeks (fitness, body composition, EF, and mental health). Open-label with blinded outcome assessors/analysts; intention-to-treat (ITT) with multiple imputation.

**Results:**

At 8 weeks, data were available for 154 participants (IG 78; CG 76); all 160 were analyzed per ITT. Compared to the CG, the IG demonstrated significantly higher mean levels in all primary PA outcomes over 8 weeks (daily steps: mean 10,356, SD 1245 versus 8242, SD 1087; Δ=2114; *d*=1.81, 95% CI 1.44‐2.18; *P*<.001; daily MVPA: mean 71, SD 15 versus 43, SD 12 min; Δ=28 min; *d*=2.06, 95% CI 1.68‐2.45; *P*<.001; and weekly MET-min: mean 1650, SD 310 versus 1340, SD 285; Δ=310; *d*=1.04, 95% CI 0.71‐1.37; *P*<.001). Adherence was also higher in the IG (both *P*<.001). Key secondary outcomes, including skeletal muscle mass (Δ +0.54 kg), body fat percentage (Δ −0.46 percentage points), 800/1000 m run time (Δ −6.5 s), and depressive symptoms (Δ −2.0 points), significantly favored the IG (all *P*<.001). No serious adverse events occurred.

**Conclusions:**

This RCT demonstrated that integrating gamification into an mHealth platform significantly enhanced PA, intervention adherence, and selected health outcomes among college students. The key innovation lies in the trial design, which held the technology platform and activity goals constant across groups, thereby allowing a clearer estimate of the incremental contribution of gamification. Unlike many existing studies, this approach reduces confounding from co-occurring intervention components in between-group comparisons. It therefore provides more comparable and methodologically robust evidence on the specific efficacy of gamification in mHealth interventions. For real-world implementation, this model leverages the existing smartphone and wearable ecosystem, offering a low-cost, scalable strategy for university health-promotion programs.

## Introduction

Physical inactivity has become a prevalent issue among today’s college student population, posing a significant threat to both their physical and mental well-being as well as their overall quality of life. Recent national estimates indicate that the combined prevalence of overweight and obesity among Chinese adults aged ≥18 years exceeds 50% [1], with college students being particularly affected. This cluster of risks, including insufficient physical activity (PA), imbalanced diet, excess weight, poor sleep, and psychological distress, not only undermines individual development but also poses challenges to national public health policies, such as the “Healthy China 2030” and “Weight Management Year” campaigns [2,3].

Traditional exercise programs often underperform because they are monotonous, provide delayed feedback, and lack sustained motivational scaffolding, which collectively erode adherence and limit scalability [4]. By contrast, mobile health (mHealth) leverages smartphones, wearables, and connected data to enable real-time, personalized monitoring and feedback on activity, heart rate, sleep, and related indicators [5]. Recent reviews and empirical studies have confirmed that mHealth interventions can produce small-to-moderate improvements in PA and related health behaviors [6]. In parallel, gamification is the purposeful use of points, badges, leaderboards, challenges, and achievement systems in nongame contexts and has gained traction in health promotion for strengthening motivation and social engagement [7]. However, evidence for the impact of gamification is mixed: while some studies report significant benefits, others show minimal effects, highlighting the need for further research on the mechanisms and optimal design of gamified interventions [8].

Self-determination theory (SDT) provides a framework for designing more effective interventions. SDT posits that satisfying the psychological needs for autonomy, competence, and relatedness supports motivation internalization and sustained behavior change. In our design, autonomy was supported via self-selection and personalized goals, competence via real-time feedback and progress dashboards, and relatedness via team-based collaboration and competition [9]. Similarly, the theory of planned behavior (TPB) contends that behavior is driven by intention, which is shaped by attitude, subjective norms, and perceived behavioral control [10].

In our gamification context, these determinants were strengthened as follows: positive outcome expectancies aimed to improve attitudes; peer norms embedded in teams and leaderboards helped establish subjective norms; and clear weekly targets were designed to enhance participants’ perceived behavioral control [11].

Recent studies and the World Health Organization (WHO) Global Action Plan on Physical Activity 2018‐2030 both emphasize that such theory-driven, cross-sectoral approaches are essential for promoting sustainable PA [12]. However, there remains a scarcity of research that systematically integrates mHealth with gamification under a combined SDT-TPB framework and rigorously evaluates its efficacy among college students. Furthermore, many existing studies rely on self-reported outcomes or focus on a narrow set of physiological measures, lacking comprehensive evaluation across physical, cognitive, and mental health domains within a single trial [13].

Integrating gamification with mHealth, therefore, offers a promising approach that combines real-time, data-driven monitoring with motivational and socially interactive elements to transform health behaviors into a more visualized, task-oriented, and engaging process. Within this framework, TPB helps explain the formation of behavioral motives and intentions, whereas SDT elucidates how short-term motivation is converted into long-term engagement [14]. This synergy is expected not only to promote PA but also to support mental well-being and, via goal setting and self-monitoring demands, to engage core domains of executive function [15]. College students, who have high smartphone penetration and digital literacy, are therefore an ideal population for such an approach [16].

Therefore, the aim of this study was to evaluate the feasibility and effectiveness of a novel, theory-based gamified mHealth intervention incorporating an incentive mechanism. This intervention integrates real-time monitoring, team-based competition, and personalized feedback and was comprehensively evaluated using a randomized controlled trial (RCT) design [17]. We prespecified two primary outcomes, (1) PA levels and (2) intervention adherence, to capture the core efficacy of the model. Secondary outcomes included physical fitness, body composition, mental health, and executive function (EF) to further assess the potential broader benefits for physical and psychological well-being. We hypothesized that, relative to control, participants receiving the gamified intervention would demonstrate higher PA levels and better adherence after the intervention and would show more favorable changes across secondary outcomes [18]. By addressing these objectives, this study aims to provide a scientific basis and practical strategies for college health promotion in the context of the “Healthy China” initiative and contributes to the innovation of health education systems in higher education institutions.

## Methods

### Trial Design

This study used a 2-arm, parallel-group (RCT) design to evaluate the feasibility and effectiveness of a gamified, competition-based intervention using mHealth technology (intervention group [IG]) versus a conventional intervention approach (control group [CG]) in promoting physical and mental health among college students aged 18‐25 years. The formal intervention period lasted for 8 weeks. Baseline assessments (T0) were conducted one week prior to the intervention, and postintervention assessments (T1) were carried out one week after the intervention concluded.

Prior to the formal intervention, all participants underwent a 1-week preintervention phase, during which the accuracy of the wearable smartwatches was validated (tolerance ≤3%), and participants were familiarized with the “Shouti Fitness” app (Tianbao Zhu) testing procedures and measurement protocols.

The study was designed and reported in accordance with the CONSORT (Consolidated Standards Of Reporting Trials) 2025 statement for randomized trials, the CONSORT-EHEALTH (Consolidated Standards of Reporting Trials of Electronic and Mobile Health Applications and Online Telehealth; Checklist 1) extension for web- and mobile-based health interventions, and the CONSORT extension for abstracts [19-21].

### Trial Setting and Eligibility Criteria

Participants were recruited between February 04 and 10, 2025, at Yantai University, Shandong Province, through campus announcements, social media platforms, and health education lectures. A total of 224 volunteers were initially screened. Eligibility criteria were the following: full-time college students aged 18‐25 years owning a smartphone; BMI between 18.5 and 30.0; and willingness to provide written informed consent. Exclusion criteria included the presence of severe physical or mental illnesses or contraindications to exercise; participation in other PA interventions within the past 3 months; and the use of psychotropic medications at the time of this writing. After screening, 160 eligible participants were enrolled.

### Eligibility Criteria for Sites and Individuals Delivering the Interventions

No eligibility criteria were prespecified for individuals delivering the interventions. The interventions were primarily technology-mediated and self-administered by participants. The role of the research team was limited to initial instruction, technical support, and data monitoring, rather than direct delivery of the exercise intervention itself.

### Intervention and Comparator

The intervention system comprised the following hardware and software components.

**Figure 1.**
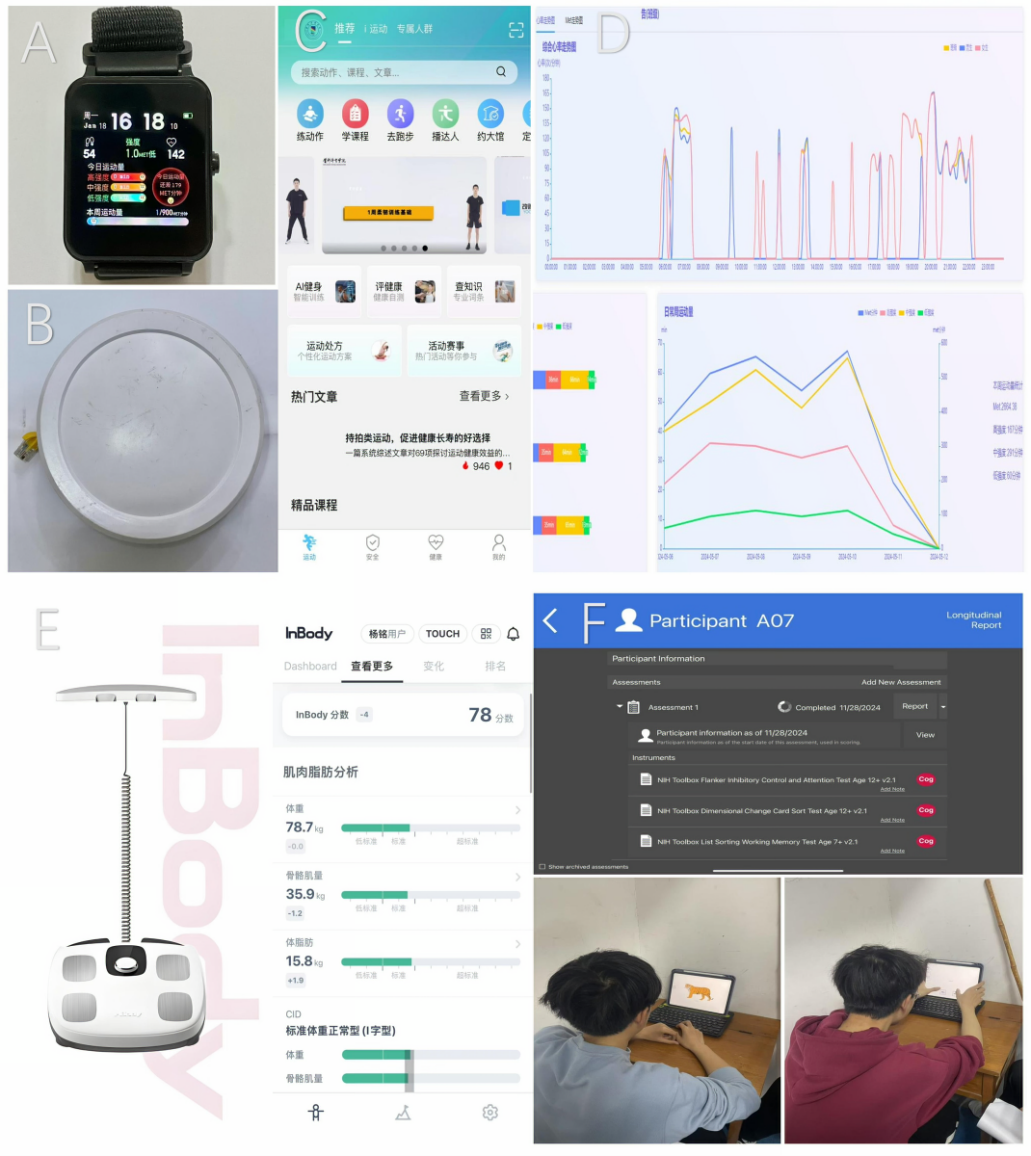
Technical platform used in this study. (**A**) High-precision fitness watch. (**B**) Data transmission station. (**C**) “Shouti Fitness” App. (**D**) Health behavior monitoring system. (**E**) InBody H30 body composition analyzer. (**F**) NIH Toolbox cognitive function assessment tool on the iPad platform.

- Hardware: participants wore fitness watches equipped with high-precision accelerometers to monitor key metrics such as step count (Figure 1A-1B), duration of moderate-to-vigorous physical activity (MVPA), and metabolic equivalent (MET).
- Software: a customized mobile app, the “Shouti Fitness” app (compatible with Android and iOS; Figure 1C ), was employed. The app features AI-driven exercise guidance, personalized exercise prescription delivery, real-time feedback, and synchronization of PA data, supporting both intervention implementation and adherence monitoring (Figure 1D).

**Figure 2.**
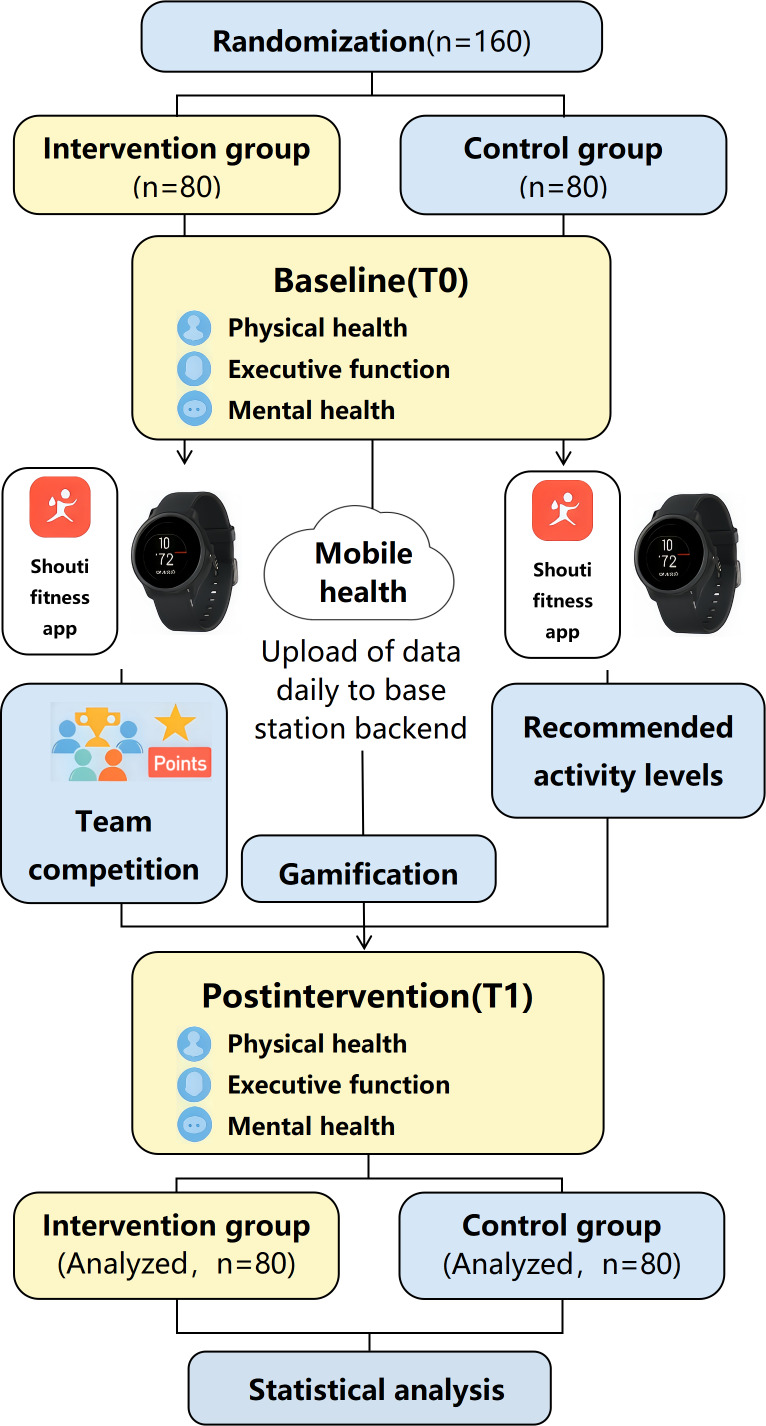
Workflow diagram of the intervention mechanism used in this study. Overview of the 8-week randomized controlled trial (RCT) workflow in college students, including T0/T1 assessments, physical activity targets, and intervention group-only gamified components.

All participants were instructed to download and register on the “Shouti Fitness” app and were provided with a study-issued fitness watch. They then completed a 1-week familiarization phase (February 17‐23, 2025) to learn the procedures and adapt to the device. The formal intervention ran for 8 weeks, from March 3 to April 27, 2025 (Figure 2).

During the intervention, the fitness watches estimated exercise intensity and energy expenditure (metabolic equivalent-minutes [MET-min]) using a convolutional neural network-long short-term memory‌ (CNN-LSTM) model and recorded durations of MVPA [22]. Participants were required to complete daily recommended PA targets (≥30 minutes of MVPA or ≥180 MET-min), with weekly goals set at ≥150 minutes of MVPA or ≥900 MET-min [23]. Data synchronization occurred twice daily at fixed data stations (07:30 AM and 11:30 PM), with activity data automatically uploaded to the exercise behavior monitoring system.

In IG (n=80) participants were assigned to 4 teams, 2 single-sex teams (1 all-male or 1 all-female), and 2 mixed-sex teams (10 males and 10 females each). Beyond meeting the baseline activity goals, a team-based competition was implemented; weekly rankings of teams based on average activity levels awarded points as follows. 1st place: 40 points, 2nd: 30 points, 3rd: 20 points, and 4th: 10 points. Additionally, the top 10% of individuals within each team (overall top 8 performers) contributed an extra 20 points to their team. Real-time activity progress bars (MVPA duration and MET-min) were displayed via the fitness watch and monitoring platform. Weekly midweek updates announced leading teams and individual champions every Wednesday, with complete team and individual leaderboards published each Sunday. Feedback and incentives included cumulative volunteer hours, honorary certificates, academic credit coordination, and material rewards of 400 Chinese yuan (Renminbi; approximately US$55.53 or €49.31, based on the exchange rate in April 2025) for individuals ranking in the top 10%.

CG (n=80) received an active, technology-based PA program without any gamification or social competition. Participants used the same technical platform, fitness watches, and app system as the IG and received identical daily and weekly personalized activity goal prompts. This design controlled for the nonspecific effects of platform use, goal setting, and researcher attention. The key distinction was the absence of all gamification and team-based competition elements, including team assignments; the point-based scoring/ranking system; real-time progress bars; midweek updates; and weekly leaderboards; as well as any performance-based feedback, incentives, or rewards.

### Outcomes

The outcome measures were prespecified as primary and secondary. Primary outcomes directly assess the core efficacy of the mHealth-gamification intervention; secondary outcomes examine potential downstream health benefits.

#### Primary Outcomes

The primary outcomes were PA levels and intervention adherence. These indicators are selected to test the core hypothesis of this study, that “mHealth+gamification” intervention model would effectively increase participants’ PA and adherence.

- PA levels: daily steps, daily minutes of MVPA, and weekly MET-min were derived from the self-developed wearable device and a companion sports application. Intensity classification followed contemporary MET cut-points (moderate 3.0‐5.9 METs; vigorous ≥6.0 METs), and weekly behavioral targets were benchmarked to the 2020 WHO adult guidelines (≥150‐300 min per week of moderate activity, or ≥75‐150 min per week of vigorous activity, or an equivalent combination) [23].
- Adherence: calculated from the health behavior monitoring system, including daily MVPA≥30 min or MET-min≥180; weekly MVPA≥150 min or MET-min≥900, aligning with WHO guidelines [23]. The data synchronization rate was the percentage of weeks with successful device-data synchronization on ≥5 days. Group-level results are presented as the mean (SD) of these individual percentages.

#### Secondary Outcomes

The secondary outcomes evaluated the potential far-end health benefits of increased PA, including physical fitness, mental health, EF, and body composition. We prespecified mental health and EF as secondary outcomes because (1) increasing PA is associated with lower depressive and anxiety symptoms in adult and college populations, and exercise shows small-to-moderate benefits in randomized trials [24] and (2) team-based, goal-driven gamification requires planning, monitoring, inhibition, and cognitive flexibility, core executive domains plausibly sensitive to change during an 8-week intervention [25].

- Physical fitness: physical fitness was field-tested on the Changxiang 100 platform in accordance with the National Student Physical Fitness Standards (GB/T 18041‐2022). The assessment comprised 7 tests to evaluate multiple facets of physical fitness: 1000 m run (men) / 800 m run (women), vital capacity, sit-and-reach test, 1-min sit-ups (women) / pull-ups (men), 50 m sprint, and standing long jump. This standardized national protocol ensures the assessments’ widespread applicability and comparability.
- Mental health: depressive and anxiety symptoms were assessed online (Wenjuanxing) using the Zung Self-Rating Depression Scale (SDS) and the Zung Self-Rating Anxiety Scale (SAS). These scales are well-established tools with demonstrated good reliability and validity (eg, SAS Cronbach *α*=.84; SDS Cronbach *α*=.88). Recent studies in Chinese student samples and broader Chinese cohorts support acceptable internal consistency and the applicability of Chinese norms for SDS/SAS. The SDS comprises 20 items, each rated on a 4-point scale, with the total raw score converted to an index. Similarly, the SAS contains 20 items rated on a 4-point scale. For both scales, higher standardized scores indicate a greater severity of symptoms. We report raw and standardized scores; higher scores indicate greater symptom burden [26].
- EFs: EFs were measured on iPad using the National Institutes of Health (NIH) Toolbox Cognition Battery (Figure 1F). This computerized assessment battery was selected due to its strong psychometric properties, standardization, and ability to efficiently measure core components of EF. The battery specifically targeted 3 key domains: the dimensional change card sort test, which assesses cognitive flexibility by requiring individuals to shift between matching rules; the flanker inhibitory control and attention test, which measures inhibitory control and attention by assessing the ability to suppress responses to distracting stimuli; and the List Sorting Working Memory Test, which evaluates working memory capacity through the temporary storage and manipulation of information. Scoring used age-adjusted T-scores per the latest NIH Toolbox V3 technical guidance (renormed to the 2020 US Census and validated against gold-standard measures). Recent meta-analyses and reviews indicate that persistent PA and movement-based programs can yield small-to-moderate improvements in EF in youth and young adults, especially in inhibitory control and cognitive flexibility; acute-exercise effects are mixed, underscoring the focus on multiweek training [27].
- Body composition: body fat percentage, skeletal muscle mass, BMI, and basal metabolic rate (BMR) were obtained with a multifrequency bioelectrical impedance analyzer (InBody H30 (InBody Co, Ltd; Figure 1E). We adhered to standard pretest controls (eg, no vigorous exercise or large meals prior to testing). This objective bioelectrical impedance analysis ensured accurate tracking of changes in body composition resulting from the PA intervention.

### Sample Size

Based on findings from previous comparable studies [28-30], the sample size was determined using G*Power 3.1 (Heinrich Heine University Düsseldorf). A 2-tailed significance level of *α*=.05, statistical power (1-*β*)=.80, and an anticipated medium effect size (*d*=0.5) were specified. For an independent samples 2-tailed *t* test comparing 2 groups, using a 2-tailed test, the minimum required sample size was 64 participants per group (total N=128). Allowing for an estimated 20% attrition, the final target was set at 80 participants per group (total N=160).

### Randomization

Random allocation sequences were generated using the PROC PLAN procedure in SAS 9.4 (SAS Institute Inc) by an independent researcher who was not involved in participant recruitment, outcome assessment, or data analysis. We used sex-stratified block randomization with a block size of 4 and a 1:1 allocation ratio to the IG (n=80) and CG (n=80) to ensure balance in sex distribution. Allocation concealment was maintained using sequentially numbered, opaque, sealed envelopes prepared off-site; after completion of baseline assessments, research staff opened the next envelope in sequence to assign participants to groups. Recruitment staff had no access to the allocation sequence; assignment was revealed when the next sealed envelope was opened after baseline assessments.

For the IG only, a postrandomization team structure was created to implement the gamified competition mechanics. The IG was subdivided into 4 equal-size teams (n=20 each). An independent coordinator used constrained random allocation based only on sex and headcount (no outcome data) to form 2 single-sex teams (Team A: 20 males; Team B: 20 females) and 2 mixed-sex teams (Teams C-D: 10 males and 10 females each). Each team functioned as an independent gamification unit for scoring and ranking. In contrast, CG participants were not assigned to any teams and did not participate in gamified competitions; they only completed the baseline PA tasks set on the same platform. This subdivision served two purposes: (1) to create the stable in-app subgroups required for points, leaderboards, and weekly challenges and (2) to promote competition fairness and mitigate structural influences arising from sex-related differences in PA and fitness [31,32].

### Blinding

Given the visible nature of the intervention components, namely the gamified team competition and group structure, this study adopted an open-label design, and participant blinding was not feasible. Participants were informed that they would be randomly assigned either to the IG, which involved team-based gamified competition, or to the CG, which involved only meeting the basic PA targets. Outcome assessors were blinded. Physical-fitness testers, InBody H30 technicians, and NIH Toolbox/SDS/SAS administrators used deidentified testing schedules, were trained not to discuss allocation, and logged any accidental unmasking. Data analysts worked on a deidentified dataset with groups masked as A/B; the statistical analysis plan was finalized and code frozen before unmasking, which occurred only after database lock and completion of primary analyses.

### Statistical Methods

Data analysis followed the intention-to-treat (ITT) principle. Continuous variables were expressed as mean (SD), and categorical variables as frequency and percentage (n%). Baseline differences were assessed using independent-samples *t* tests (or Mann-Whitney *U* tests, as appropriate) for continuous variables and *χ*² tests (or Fisher exact tests) for categorical variables.

The primary outcomes (PA and adherence) were summarized as mean values over the 8-week intervention period. Between-group differences in these postintervention means were then assessed using ANCOVA, with the corresponding baseline values included as covariates. For secondary outcomes (physical fitness, body composition, SDS/SAS, and EF), ANCOVA with baseline adjustment was likewise used, with mixed effects models as sensitivity analyses. Adherence-related indicators (eg, goal completion and data synchronization) were summarized descriptively and compared between groups; when applicable, ANCOVA with baseline adjustment was applied. Sex-based comparisons were not prespecified, and the trial was not powered for sex-related effect modification. To avoid multiplicity and over-interpretation, no inferential subgroup analyses (eg, group×sex interactions) were conducted; inference focuses on overall between-group effects.

To address missing data, Little test for missing completely at random (MCAR) was applied to all outcomes measured at T0 and T1. The results showed no significant departure from MCAR (*χ*²_15_=11.236; *P*=.73). Given the low proportion of missing values (<5%) and to preserve statistical power and reduce potential bias associated with listwise deletion, multiple imputation (m=20) using chained equations was performed under a missing-at-random assumption. The imputation model included group assignment (IG vs CG), sex, age, baseline values of each outcome, and adherence indicators (eg, percentage of days meeting the PA goal) as predictors. Robustness was confirmed by reporting both complete-case and multiple imputation–pooled estimates for primary outcomes and through sensitivity analyses that excluded algorithm-flagged suspect data, which yielded consistent conclusions. All statistical tests were 2-sided, with *P*<.05 considered statistically significant.

Analyses were performed using SPSS (version 26.0; IBM Corp).

### Ethical Considerations

The study received ethics approval (Approval No. 2025A125, approved on January 15, 2025) from the Ethics Committee of Capital University of Physical Education and Sports. The study protocol was finalized before the start of participant recruitment (February 4, 2025) and is identical to the protocol retrospectively registered in the Chinese Clinical Trial Registry (ChiCTR2500108950) with respect to trial design, eligibility criteria, prespecified primary and secondary outcomes, and planned statistical analyses. No changes were made to these elements after trial commencement.

Trial registration was completed retrospectively in the Chinese Clinical Trial Registry (ChiCTR2500108950) on September 9, 2025, after enrollment had begun, because prospective registration was inadvertently not completed during trial setup due to an administrative oversight; this was not an attempt to avoid registration. To mitigate concerns about selective reporting, the institutional review board (IRB)–approved protocol was finalized prior to recruitment and remained unchanged throughout the trial with respect to the trial design, eligibility criteria, prespecified outcomes, and planned statistical analyses. An official letter from the IRB confirming that the submitted study design is identical to the protocol assessed and approved before trial initiation has been provided to the editorial office for review.

All procedures were conducted in accordance with the principles of the Declaration of Helsinki. Before enrollment, all participants received written and verbal information about the study purpose, procedures, potential risks, and anticipated benefits and subsequently provided written informed consent. Participants were informed that participation was voluntary and that they could withdraw from the study at any time without providing a reason. This withdrawal would incur no penalty or detriment to their academic standing. To ensure privacy and confidentiality, all participant data were deidentified upon collection and assigned a unique study ID. The analytical dataset contained only these anonymized IDs. The master list linking IDs to personal information was stored separately on a secure, password-protected server with access restricted to the principal investigator and the data manager. Participants who completed the entire intervention cycle received nonmonetary compensation (accumulated volunteer hours, a certificate of honor, and coordination with academic credit where applicable). Additionally, the top 10% of performers received a material reward of 400 Chinese yuan (Renminbi; approximately US $55.53 or €49.31, based on the exchange rate in April 2025). No identifiable images of participants are presented in this manuscript or in the supplementary materials; the participants pictured in Figure 1F provided written consent for publication of the deidentified photographs for academic purposes.

### Data Security and Risk Management

To prevent cheating and ensure data integrity, each smartwatch (serial number/MAC) was identity-verified and bound 1:1 to a unique participant account. Weekly, a random 10% sample of participants underwent raw-data audits in which 7-day device traces (steps, moderate-to-vigorous physical activity [MVPA], metabolic equivalent-minutes [MET-min], and wear time with timestamps) were cross-checked against server sync logs; discrepant days were documented and flagged for sensitivity analyses. For privacy and confidentiality, analytical datasets were deidentified, access followed role-based access control under a least-privilege policy, and data were used solely for academic research as approved by the ethics committee; all access and exports were audit-logged. To reduce attrition, participants received a standardized briefing on study requirements before randomization; withdrawals were allowed at any time, and reasons were recorded and summarized in the CONSORT flow.

### Harms

Safety was monitored through weekly checks and audits using predefined triggers; events were logged by a blinded assessor. Serious adverse events required immediate IRB reporting and discontinuation as prespecified in the protocol. Patients or members of the public were not involved in this trial. No important changes to the trial protocol, outcomes, or analyses were made after trial initiation.

## Results

### Participant Flow

**Figure 3.**
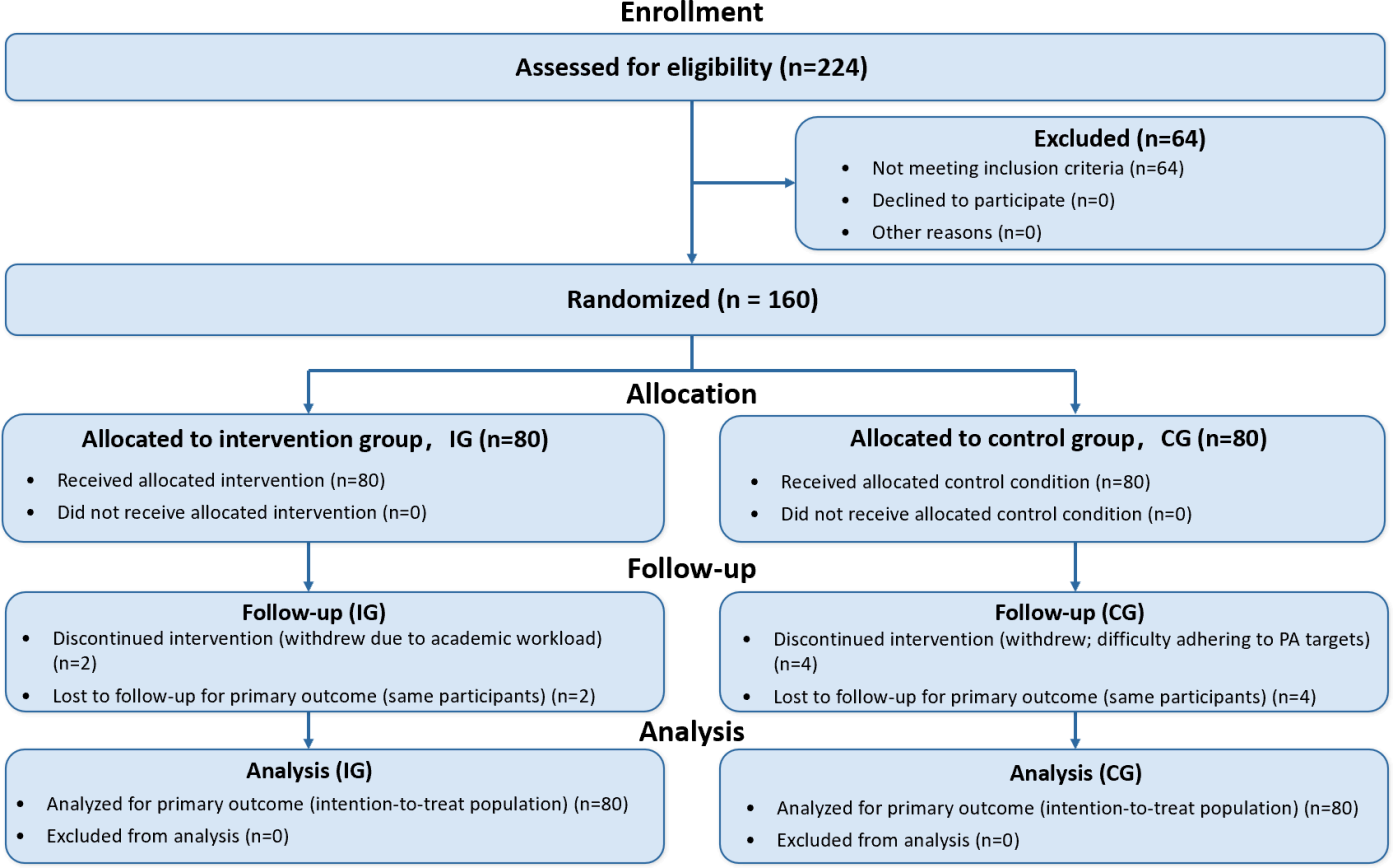
CONSORT (Consolidated Standards of Reporting Trials) flow diagram, participant flow through the randomized trial. CG: control group; IG: intervention group; PA: physical activity;

A total of 224 volunteers were screened for eligibility. After excluding 64 individuals who did not meet the inclusion criteria, 160 participants were enrolled and randomly assigned in a 1:1 ratio to the IG and CG, with 80 participants in each group (Figure 3).

During the 8-week intervention period, 6 participants withdrew from the study (IG: 2 participants, 2.5%; CG: 4 participants, 5%). The primary reasons for dropout were academic workload (n=2) and failure to meet PA targets (n=4). Overall, this resulted in 3.8% missingness in post-intervention outcomes. Little MCAR test across all primary and secondary outcomes indicated that this missingness did not significantly depart from MCAR (*χ*²_15_=11.236; *P*=.73).

The trial ended as planned after completion of the prespecified 8-week intervention and postintervention assessments and was not stopped early.

### Baseline Data

There were no statistically significant differences between the 2 groups in baseline demographic characteristics (age, sex, and BMI) or psychological scale scores (*P*>.05), indicating good comparability between group (Table 1).

**Table 1.** Baseline characteristics of participants.

Characteristic	IG^a^ (n=80)	CG^b^ (n=80)	Total (N=160)	*P* value
Age (years), mean (SD)	20.5 (1.2)	20.8 (1.3)	20.6 (1.3)	.15
Sex, n (%)				
Female	40 (50.0)	40 (50.0)	80 (50.0)	>.99
Male	40 (50.0)	40 (50.0)	80 (50.0)	>.99
Body composition, mean (SD)				
Body weight, (kg)	66.4 (12.5)	65.8 (11.8)	66.1 (12.1)	.76
BMI (kg/m²)	22.4 (2.5)	22.1 (2.3)	22.2 (2.4)	.42
Mental health, mean (SD)				
SAS^c^ score	44.3 (7.2)	45.1 (5.9)	44.7 (6.6)	.43
SDS^d^ score	46.4 (7.7)	45.9 (8.1)	46.2 (7.9)	.67
PA^e^				
Daily steps, mean (SD)	7842 (1,603)	7921 (1,425)	7882 (1,514)	.74
MVPA^f^ (min/day), mean (SD)	37.2 (11.5)	37.4 (10.7)	37.3 (11.1)	.91
Weekly MET-min^g^, mean (SD)	1020 (205)	1035 (220)	1028 (212)	.66

a IG: intervention group.

b CG: control group.

c SAS: Self-Rating Anxiety Scale.

d SDS: Self-Rating Depression Scale.

e PA: physical activity.

f MVPA: moderate-to-vigorous physical activity.

g MET-min: metabolic equivalent-minute.

### Intervention and Comparator Delivery

Both arms were technology-mediated and primarily self-administered; the study team provided standardized onboarding, technical support, and data monitoring. No crossovers or major deviations from the intended delivery were identified, and adherence indicators (goal completion and data synchronization) are summarized in Table 2.

**Table 2.** Outcome during the 8-week intervention and change from T0 to T1.

Domain and outcome^a^		IG^b^ (n=80), mean (SD)	CG^c^ (n=80), mean (SD)	Δ (IG-CG)	Δ IG-CG (%)	*P* value	Effect size, *d^d^* (95% CI)
PA^e^ levels^f^							
Daily steps		10,356 (1245)	8242 (1087)	+2114	25.6	<.001	1.81 (1.44 to 2.18)
Daily MVPA^g^ (min)		71 (15)	43 (12)	+28	65.1	<.001	2.06 (1.68 to 2.45)
Weekly MET-min^h^		1650 (310)	1340 (285)	+310	23.1	<.001	1.04 (0.71 to 1.37)
Adherence (%)							
Goal completion rate		88.9 (8.5)	77.5 (10.2)	+11.4	14.7	<.001	1.21 (0.88 to 1.55)
Data sync rate		89.1 (7.7)	75.5 (11.3)	+13.6	18.0	<.001	1.41 (1.06 to 1.75)
Body composition							
Δ Skeletal muscle (kg)		+0.86 (0.23)	+0.32 (0.11)	+0.54	168.8	<.001	3.00 (2.54 to 3.45)
Δ Body fat (%)		−1.46 (0.51)	−1.00 (0.32)	−0.46	46.0	<.001	−1.08 (−1.41 to −0.75)
Δ BMR^i^ (kcal/d)		+20.7 (4.9)	+12.9 (4.8)	+7.8	60.5	<.001	1.61 (1.25 to 1.97)
Δ Body weight (kg)		−1.17 (1.45)	−0.91 (1.45)	−0.26	28.6	.26	−0.18 (−0.49 to 0.13)
Δ BMI (kg/m²)		−0.45 (1.10)	−0.26 (1.04)	−0.19	73.1	.26	−0.18 (−0.49 to 0.13)
Physical fitness							
Δ 800/1000 m run (s)		−13.0 (6.0)	−6.5 (4.0)	−6.5	100.0	<.001	−1.27 (−1.62 to −0.93)
Δ Vital capacity (mL)		+470 (110)	+280 (80)	+190	67.9	<.001	1.98 (1.60 to 2.35)
Δ Sit-and-reach (cm)		+5.2 (1.1)	+3.4 (1.2)	+1.8	52.9	<.001	1.56 (1.21 to 1.92)
Δ Sit-ups (female), counts (n=40 per group)		+10.5 (2.0)	+6.0 (1.5)	+4.5	75.0	<.001	2.55 (1.95 to 3.14)
Δ Pull-ups (male, counts, n=40 per group)		+0.6 (0.5)	+0.4 (0.5)	+0.2	50.0	.08	0.40 (−0.04 to 0.84)
Δ 50 m sprint (s)		−0.30 (0.30)	−0.25 (0.30)	−0.05	20.0	.28	−0.17 (−0.48 to 0.14)
Δ Standing long jump (cm)		+8.5 (13.1)	+4.5 (14.5)	+4.0	88.9	.07	0.29 (−0.02 to 0.60)
EF^j^							
Δ Cognitive flexibility (ms)		−104 (26)	−75 (32)	−29	38.7	<.001	−0.99 (−1.32 to −0.67)
Δ Inhibitory control (errors)		−1.4 (2.5)	−1.0 (2.5)	−0.4	40.0	.31	−0.16 (−0.47 to 0.15)
Δ Working memory (correct)		+0.5 (1.5)	+0.3 (1.3)	+0.2	66.7	.38	0.14 (−0.17 to 0.45)
Mental health							
Δ SDS^k^ (depression)		−4.5 (2.0)	−2.5 (1.5)	−2.0	80.0	<.001	−1.13 (−1.47 to −0.80)
Δ SAS^l^ (anxiety)		−2.2 (3.5)	−1.3 (3.1)	−0.9	69.2	.09	−0.27 (−0.58 to 0.04)

a Values are mean (SD). Analyses followed intention-to-treat with multiple imputation

b IG: intervention group.

c CG: control group.

d Effect sizes are Cohen *d* (pooled SD); the sign of *d* follows Δ (IG−CG).

e PA: physical activity.

f PA and adherence outcomes were summarized over the 8-week intervention period; outcomes prefixed with Δ are change scores (T1−T0).

g MVPA: moderate-to-vigorous physical activity.

h MET-min: metabolic equivalent-minutes.

i BMR: basal metabolic rate.

j EF: executive function.

k SDS: Self-Rating Depression Scale.

l SAS: Self-Rating Anxiety Scale.

No concomitant care was provided by the study team, and concomitant interventions outside the trial that could influence outcomes were not systematically monitored.

### Numbers Analyzed

Primary and secondary outcomes were analyzed according to the ITT principle (IG n=80; CG n=80). Missing postintervention (T1) values were handled using multiple imputation as prespecified. Outcome data were available at T1 for 78/80 participants in the IG (97.5%) and 76/80 in the CG (95.0%). Table 2 presents MI-pooled descriptive statistics and between-group inferences based on the primary ITT analysis.

### Outcomes and Estimation

#### Primary Outcomes

##### PA Levels

**Figure 4.**
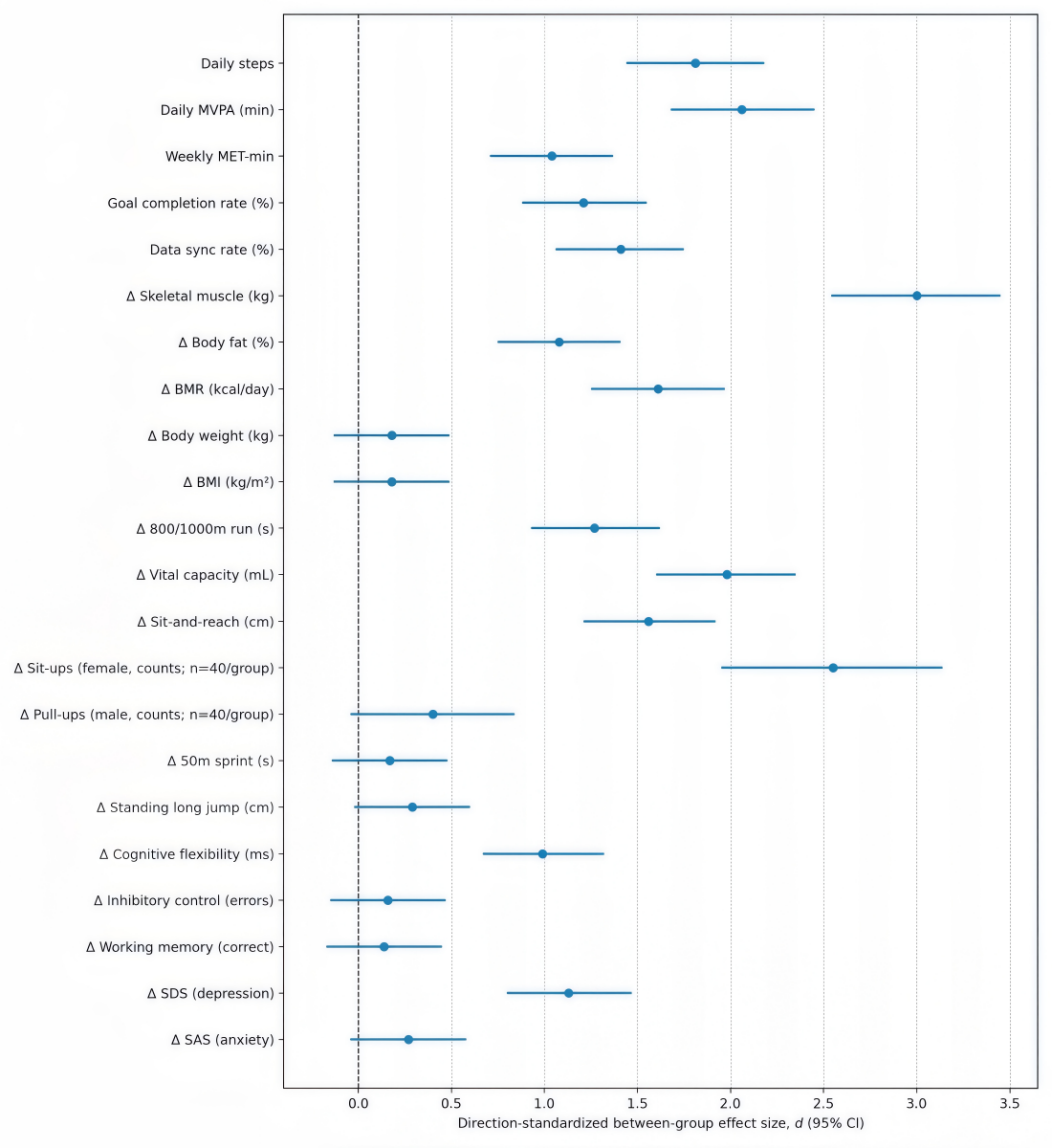
Effect sizes (*d*) and 95% CIs for each outcome measure (IG vs CG): positive values favor IG (directions standardized). The dashed vertical line indicates no between-group difference (*d*=0); CIs crossing 0 indicate nonsignificance. MET-min: metabolic equivalent-minute; MVPA: moderate-to-vigorous physical activity; SAS: Self-Rating Anxiety Scale; SDS: Self-Rating Depression Scale.

Across the 8-week period, both arms showed the typical early rise-mid plateau-late rebound pattern. Adjusted between-group differences consistently favored IG for steps, daily MVPA, and weekly MET-min throughout the intervention (all *P*<.001). These results indicate that the gamified mHealth arm elicited additional PA beyond baseline platform targets; see Table 2 for detailed outcomes and 95% CIs and Figure 4 for effect sizes.

##### Intervention Adherence

IG demonstrated significantly higher adherence—for both goal completion and data synchronization—than CG (all *P*<.001). Exact means, between-group differences (Δ), and effect sizes with 95% CIs are reported in Table 2.

### Secondary Outcomes

#### Physical Fitness

The intervention produced differential effects across fitness levels. For physical fitness, IG improved more than CG in aerobic endurance (800/1000 m run), vital capacity, muscular endurance (female sit-ups), and flexibility (sit-and-reach) (all *P*<.001), while no between-group differences emerged for 50 m sprint, pull-ups, or standing long jump (*P*>.05). See Table 2 and Figure 4 for detailed outcomes and 95% CIs.

#### Body Composition

Relative to CG, IG showed larger body-composition gains, higher skeletal muscle mass, lower body fat percentage, and higher BMR (all *P*<.001), while changes in body weight and BMI showed no significant between-group differences (*P*>.05). See Table 2 for detailed outcomes and 95% CIs and Figure 4 for effect sizes.

#### EF

EF showed a domain-specific pattern, cognitive flexibility improved in IG (*P*<.001), whereas working memory and inhibitory control showed no between-group differences (*P*>.05). See Table 2 and Figure 4 for detailed outcomes and 95% CIs.

#### Mental Health Status

Depressive symptoms (SDS) decreased more in IG than in CG (*P*<.001), whereas anxiety (SAS) changes were small and not different between group (*P*>.05). Individual trajectories varied; see Table 2 and Figure 4 for detailed outcomes and 95% CIs.

### Ancillary Analyses

Given the lack of prespecification for sex-based subgroup inference, no inferential sex comparisons are reported; primary conclusions rely on overall effects.

### Harms

No study-related serious adverse events; no device-related dermatologic events requiring care; and no major technical failures or data losses affecting outcome interpretation were recorded.

## Discussion

### Principal Results

This RCT evaluated the incremental effect of adding team-based gamification and incentives to a standard wearable- and app-based PA program for college students. Compared with an active control using the identical platform and activity goals, the IG showed greater improvements in the prespecified primary outcomes—objectively measured PA and intervention adherence—and in selected secondary outcomes related to physical fitness and body composition, whereas effects on EF and anxiety outcomes were limited. This overall pattern is consistent with meta-analytic evidence that gamified mHealth interventions tend to increase daily steps/MVPA and yield small-to-moderate improvements in adiposity-related indicators compared with nongamified comparators [33,34]. Notably, by holding the app-wearable platform and activity goals constant across groups, this study may help isolate the incremental contribution of gamification and reduce confounding that is common when platforms and components differ across conditions [35]. The findings indicate that the added value of gamification in this context may operate primarily through strengthening adherence and engagement, leading to downstream benefits for selected physical outcomes [36].

### Mechanisms

Improvements in PA and adherence may be interpreted within SDT and TPB, in that need-supportive gamification could facilitate motivational internalization and help translate intention into action [37]. First, SDT posits that autonomy, competence, and relatedness are core components of intrinsic motivation [38,39]. In this study, team-based competition, real-time feedback, and visible progress cues may have supported competence and relatedness while maintaining task structure to promote more sustained engagement in PA.

In addition, the intervention combined support for basic psychological needs (autonomy, competence, and relatedness) with extrinsic incentives (points and leaderboards) [40]. From an SDT perspective, a key issue is how such external incentives are framed and delivered [41]. When points and leaderboards are presented as informational signals of personal progress rather than controlling demands, they may better support autonomy and align with the regulatory continuum of motivational internalization [42], which could help explain the observed improvements in adherence during the intervention period.

More specifically, short-term gains in PA and adherence may reflect a synergy between extrinsic prompts and need-supportive features: the former may catalyze initial participation and short-term persistence, whereas the latter may enhance self-efficacy and belonging by satisfying competence and relatedness needs [43]. Nevertheless, the salience and material nature of certain incentives may slow internalization toward more integrated regulation [44]. In this RCT, in addition to designing nonmaterial rewards such as cumulative volunteer hours, honorary certificates, and academic credit coordination, we also incorporated a conditional monetary reward for the IG, tied to leaderboard performance. This external economic incentive served as a salient extrinsic goal and could bolster adherence and help sustain PA, particularly during the mid-to-late stages of the intervention. However, its effect may attenuate after the incentive is withdrawn and could further limit motivational internalization [45]. Such incomplete internalization could partly limit the transfer of benefits to domains that typically require greater cognitive engagement, longer accumulation, or additional components beyond increasing activity volume (eg, strength-related fitness and selected EF domains) [46]. Evidence from multi-arm randomized trials directly comparing gamification, financial incentives, and their combination supports the plausibility that each component can increase PA and that combining them may yield larger improvements than either component alone, consistent with additive or synergistic effects [47]. Therefore, the intervention benefits observed in the present study are more likely the result of the combined action of the engaging environment created by gamification and the salient extrinsic goals set by monetary rewards.

Overall, the SDT-based account primarily addresses motivation formation and internalization. TPB provides a complementary perspective by clarifying how attitudes, subjective norms, and perceived behavioral control jointly shape the translation of behavioral intention into actual behavior [48]. In the present intervention, digital reminders, social-comparison signals, and personalized feedback may have strengthened perceived control and normative support, thereby facilitating daily goal execution and adherence [49].

### Interpretation and Boundaries

Unlike prior physical fitness–promotion interventions that typically report small-to-moderate effect sizes [7], the present mHealth-based intervention with added gamification showed comparatively large standardized effect sizes for objectively measured physical activity and selected fitness-related outcomes. This pattern may reflect the intervention’s efficacy under relatively controlled trial conditions. First, the single-university student cohort was relatively homogeneous in age range and daily routines, which may reduce within-sample outcome variability and thereby inflate standardized effect size estimates for a given raw mean difference [50]. Second, delivery was supported by a technology platform with high implementation intensity (eg, frequent monitoring, scheduled synchronization, and staged feedback) and high adherence during the intervention period (Table 2), which likely improved intervention fidelity and strengthened the effective “dose contrast” between groups. Prior evidence suggests that higher engagement/adherence in digital interventions is associated with larger behavioral effects [51]. However, when such interventions are scaled from controlled trial settings to more heterogeneous real-world populations with lower monitoring/support intensity, observed effect sizes may attenuate [52]. Building on this overall interpretation, we now turn to the domain-specific findings and key boundaries.

The higher frequency of MVPA in IG may have contributed to improvements in aerobic capacity [53]. This is plausible because the intervention primarily promoted cardiovascular adaptation by increasing activity volume, and the achieved activity pattern was broadly aligned with WHO-recommended PA levels [23]. In contrast, between-group differences were not statistically significant for strength- or power-related outcomes (eg, 50-m sprint, standing long jump, pull-ups). A primary explanation may lie in the structural emphasis of the intervention: the program was designed to promote MVPA via mHealth, with common modalities such as jogging and cycling that primarily load cardiorespiratory and energy-metabolic systems, but it did not include a systematic resistance-training component targeting large muscle groups [54]. Improvements in strength/power generally require more targeted resistance training, and baseline capability (eg, the ability to perform pull-ups) may also influence the magnitude of improvement [55,56].

Although the intervention did not yield statistically significant changes in body weight or BMI, IG showed significant improvements in body-composition indicators (eg, skeletal muscle mass, body fat percentage, and BMR) relative to CG. This pattern may be explained by the fact that, in the absence of strict dietary control and a standardized resistance-training dose, increasing PA can preferentially improve body composition [57]. Moreover, because adipose tissue has a lower density than muscle tissue, concurrent fat loss and muscle gain may result in a “weight substitution effect,” whereby meaningful changes in body shape occur with minimal change in total body weight [58]. In addition, reductions in fat mass together with increases in lean mass may elevate resting metabolic rate, consistent with the metabolic benefits associated with increases in fat-free mass [59].

For EF, IG showed a statistically significant improvement only in cognitive flexibility, whereas changes in working memory and inhibitory control did not reach statistical significance. This suggests that cognitive flexibility may be a domain more readily influenced by this PA-focused intervention. One possible mechanism is the acute and cumulative effects of moderate-intensity aerobic exercise on prefrontal hemodynamics and neural signaling [60]. By contrast, limited changes in working memory and inhibitory control are also consistent with the general “dose-response” and domain-specific nature of EF adaptation [61], whereby these domains may require higher intensity, longer duration, or more cognitively engaging activity to change meaningfully [62,63].

The intervention showed limited effects on anxiety symptoms, which may reflect that the protocol primarily targeted PA behavior and did not incorporate psychological components (eg, cognitive behavioral therapy, mindfulness, or stress-management modules) that more directly modulate emotional processes [64]. In addition, gamification mechanisms may be double-edged: competitive elements can enhance motivation, but they may also thwart autonomy and relatedness for some participants, potentially constraining psychological benefits [65]. Prior evidence also suggests substantial heterogeneity in mental health responses to gamified interventions, which may relate to individual sensitivity to social comparison and competitive pressure [66]. Moreover, PA tends to show stronger effects on depressive symptoms than on anxiety, and benefits may be more apparent among participants with milder baseline symptom levels, implying conditional efficacy [67].

Taken together, changes in EF and mental health may be interpreted as potential, conditional downstream benefits of PA-focused interventions rather than inevitable effects of gamification per se [62]. This further suggests that when interventions aim to target mental health or EF, it is important to align mechanisms, dose/intensity, and the target population to achieve more precise matching and maximize the likelihood of benefit [68].

### Limitations

This study used an RCT design and leveraged mHealth technology to enable real-time tracking and feedback of intervention behaviors. Nevertheless, several limitations should be acknowledged.

First, the 8-week intervention duration, while sufficient for assessing short-term efficacy, may be insufficient to observe long-term behavioral maintenance and sustained neural or physiological adaptations. Future studies with longer follow-up periods are needed to evaluate the sustainability of the effects [69]. Second, the absence of systematic control over dietary intake and sleep patterns represents a potential confounding factor for the observed changes in body composition, as these lifestyle components are known to interact with PA [70]. Third, all participants were recruited from a single university, which may limit generalizability to other collegiate populations and demographic groups; the relatively homogeneous cohort may also have reduced outcome variability [71]. Fourth, the team sex composition was implemented for fairness and feasibility rather than for testing sex differences; the study was neither powered to detect effect modification by sex, nor were such comparisons prespecified. Additionally, because conditional financial incentives were bundled with gamification only in the IG, we could not disentangle the independent effects of incentives versus gamification [72]. Moreover, although the protocol had been finalized before recruitment and remained unchanged throughout the trial, the retrospective registration of the trial due to an administrative oversight may still be considered a limitation in terms of methodological transparency and potential risk of bias. Finally, the evaluation of EF and mental health, although we cited literature [73], is limited by the lack of baseline impairment, which limits sensitivity to change and raises the possibility of ceiling effects.

### Implications

Overall, this 8-week RCT showed that, compared with a nongamified (monitoring only) intervention with identical activity goals, a gamified mHealth exercise intervention delivered within the same app-wearable system yielded clear incremental benefits: it increased PA and intervention adherence and improved selected physical fitness and body-composition indicators. The key innovation lies in quantifying the “incremental effect” attributable to gamification on top of a unified activity goal and the same technical platform, thereby allowing a clearer estimate of the incremental contribution of gamification. This approach differed from much of the prior literature, where between-group comparisons were often confounded by co-occurring intervention components [35], and it reduced such confounding by holding the technology platform and activity goals constant across groups. In terms of evaluation, by integrating objective behavioral data with a multidimensional outcome set, the study offered more robust and comparable evidence and helped delineate where gamification is most likely to be effective and where its effects may be bounded. From a real-world perspective, the model could be scaled at low cost by leveraging existing smartphone apps and wearable devices, supporting feasible implementation in university health-promotion and campus health management programs and offering an accessible digital health strategy to address physical inactivity among college students and inform related public health practice and policy [74].

### Future Directions

Building on our findings and limitations, we propose several avenues for future research: (1) investigate integration of multimodal components, such as the integration of structured resistance training components to more effectively target muscular strength and power, which showed limited improvement here [75]; (2) extend intervention exposure (eg, >12‐16 wk) and prespecify dose-response targets for PA to test whether benefits for domains like working memory and inhibition emerges with a greater cumulative dose [76]; (3) explore synergies with evidence-based psychological approaches. This includes embedding digital cognitive behavioral therapy elements or mindfulness training within the mHealth platform to directly target mechanisms underlying anxiety, depression, and cognitive regulation [77]; (4) testing the adapted intervention in more diverse populations, including individuals with baseline subclinical symptoms or lower digital literacy, will be crucial for determining its broader public health applicability [78]; (5) add pragmatic diet/sleep capture (eg, 1‐2 24-hour dietary recalls per week including one weekend day, photo-assisted intake logs, and 7-day sleep diary + actigraphy/wearable-derived sleep/heart rate) to strengthen causal attribution for body-composition outcomes [79]; (6) preregister sex-related hypotheses, power accordingly, and conduct planned interaction/subgroup analyses to evaluate potential heterogeneity of effects by sex and to align analytic decisions with study aims [80]; and (7) use multiarm or factorial designs (eg, gamification-only, incentive-only, both, and control), with postincentive follow-up, to isolate component-specific effects and assess maintenance [47].

### Conclusions

This RCT demonstrates that an mHealth intervention integrating gamification effectively enhanced physical fitness, body composition, and adherence to PA among college students. While its impact on psychological health and EF was limited, the approach demonstrates strong potential for scalable, technology-driven health promotion in young adult populations.

However, because this study did not include post-intervention follow-up, the sustainability of the effects was not assessed. Future research should evaluate maintenance and scalability in more diverse real-world settings, ideally using multiarm/factorial designs with postincentive follow-up. Studies should also test longer intervention exposure, multimodal components, and dietary strategies.

## Supplementary material

10.2196/82769 Checklist 1 CONSORT-eHEALTH checklist (V 1.6.1).

## Abbreviations

BMR: basal metabolic rate
CG: control croup
EF: executive function
IG: intervention group
IRB: institutional review board
ITT: intention-to-treat
MCAR: missing completely at random
MET-min: metabolic equivalent-minute
mHealth: mobile health
MVPA: moderate-to-vigorous physical activity
NIH: National Institutes of Health
PA: physical activity
RCT: randomized controlled trial
SAS: Self-Rating Anxiety Scale
SDS: Self-Rating Depression Scale
SDT: self-determination theory
TPB: theory of planned behavior
WHO: World Health Organization
