# Supplementary material for: A Gamified Mobile Health Intervention to Promote Physical Activity, Executive Function, and Mental Health in College Students: Randomized Controlled Trial
Source: J Med Internet Res. 2026 Apr 7;28:e82769. doi: 10.2196/82769 (PMC13055952; doi:10.2196/82769)
Supplement: Checklist 1 [file jmir-v28-e82769-s001.pdf]

CONSORT-EHEALTH (V.1.6.1) - Submission/Publication Form CONSORT-EHEALTH checklist (V.1.6.1): Information to include when reporting ehealth/mhealth trials (web-based/Internet-based intervention and decision aids, but also social media, serious games, DVDs, mobile applications, certain telehealth applications)

Section/Topic	Item No.	CONSORT* Checklist Item	EHEALTH Extensions (additions to, or clarification of the CONSORT item)	Reported on page No
**TITLE & ABSTRACT**				
	1a	Identification as a randomized trial in the title	i) Identify the mode of delivery in the title	✔
	1b	Structured summary of trial design, methods, results, and conclusions NPT: Description of experimental treatment, comparator, care providers, centers, and blinding status	i) Key features; ii) Open vs closed; iii) Use data; iv) Negative trial conclusions	✔
**INTRODUCTION**				
	2a	Scientific background and explanation of rationale	i) Problem/solution; ii) Rationale	✔
	2b	Specific objectives or hypotheses	No eHealth subitems	✔
**METHODS**				
Trial design	3a	Description of trial design (parallel, factorial, allocation ratio)	No eHealth subitems	✔
	3b	Important changes to methods after commencement	i) Bug fixes, content changes, downtimes	
Participants	4a	Eligibility criteria for participants	i) Computer/Internet literacy; ii) Recruitment mode; iii) Information during consent	✔
	4b	Settings and locations where the data were collected	i) Online vs face-to-face assessments; ii) Institutional affiliations	✔
Interventions	5	The interventions for each group with sufficient detail	i) Developers/sponsors; ii) Development process; iii) Revisions; iv) QA; v) Replicability; vi) Digital preservation; viii) Delivery/features/theory; ix) Use parameters; x) Human involvement; xi) Prompts; xii) Co-interventions	✔
Outcomes	6a	Pre-specified primary and secondary outcomes	i) Online questionnaire validation; ii) Use definition; iii) Qualitative feedback	✔
	6b	Changes to trial outcomes after commencement	No eHealth subitems	

Sample size	7a	How sample size was determined<br>NPT: clustering considerations	i) Attrition adjustment	✔
	7b	Interim analyses and stopping guidelines	No eHealth subitems	
Randomisation	8a	Method used to generate random sequence	No eHealth subitems	✔
	8b	Type of randomisation; details of restrictions	No eHealth subitems	✔
Allocation concealment	9	Mechanism to implement random allocation sequence	No eHealth subitems	✔
Implementation	10	Who generated the sequence, enrolled participants, assigned interventions	No eHealth subitems	✔
Blinding	11a	Who was blinded and how	i) Who blinded; ii) Awareness of intervention of interest	✔
	11b	Similarity of interventions	No eHealth subitems	✔
Statistical methods	12a	Statistical methods for primary/secondary outcomes	i) Missing data handling (ITT, imputation)	✔
	12b	Additional analyses	No eHealth subitems	✔
Ethics & Informed Consent	X26	(not a CONSORT item)	i) Ethics approval; ii) Consent procedures; iii) Safety/privacy	✔
**RESULTS**				
Participant flow	13a	Numbers randomized, treated, analyzed	No eHealth subitems	✔
	13b	Losses/exclusions with reasons	i) Attrition diagram	✔
Recruitment	14a	Recruitment/follow-up dates	i) Report secular events	✔
	14b	Why trial ended/stopped	No eHealth subitems	
Baseline data	15	Baseline demographic/clinical data	i) Report digital divide demographics	✔
Numbers analysed	16	Numbers in each analysis	i) Multiple denominators; ii) ITT principle	✔
Outcomes	17a	Primary/secondary outcomes, effect sizes	i) Report process outcomes, usage intensity	✔
	17b	Binary outcomes	Report absolute and relative effect sizes	✔
Ancillary analyses	18	Other analyses, subgroup/adjusted	i) Subgroup analysis of users	✔
Harms	19	All harms/unintended effects	i) Privacy/technical problems; ii) Qualitative feedback	✔
**DISCUSSION**				
Limitations	20	Trial limitations	i) Typical eHealth limitations: blinding, multiplicity, attrition bias	✔
Generalisability	21	Generalisability	i) To other populations; ii) Differences in routine use	✔
Interpretation	22	Interpretation consistent with results	i) Restate questions/answers; ii) Highlight unanswered/future research	✔

**OTHER INFORMATION**				
Registration	23	Trial registration	No eHealth subitems	✔
Protocol	24	Protocol access	No eHealth subitems	✔
Funding	25	Funding sources and roles	No eHealth subitems	✔
Competing interests	X27	(not a CONSORT item)	i) Relation of study team to system evaluated	✔

* CONSORT = Consolidated Standards of Reporting Trials

** NPT = non pharmacological treatment (CONSORT extension)

References

1. Schulz, K. F., Altman, D. G., Moher, D., & Consort Group. (2010). CONSORT 2010 statement: updated guidelines for reporting parallel group randomised trials. *Journal of clinical epidemiology*, *63*(8), 834-840.

2. Eysenbach, G., & Consort-EHEALTH Group. (2011). CONSORT-EHEALTH: improving and standardizing evaluation reports of Web-based and mobile health interventions. *Journal of medical Internet research*, *13*(4), e126.

3. Moher, D., Hopewell, S., Schulz, K. F., Montori, V., Gøtzsche, P. C., Devereaux, P. J., ... & Altman, D. G. (2010). CONSORT 2010 explanation and elaboration: updated guidelines for reporting parallel group randomised trials. *Bmj*, *340*.

4. Blankers, M., Koeter, M. W., & Schippers, G. M. (2010). Missing data approaches in eHealth research: simulation study and a tutorial for nonmathematically inclined researchers. *Journal of medical Internet research*, *12*(5), e1448.

5. Proudfoot, J., Klein, B., Barak, A., Carlbring, P., Cuijpers, P., Lange, A., ... & Andersson, G. (2011). Establishing guidelines for executing and reporting internet intervention research. *Cognitive behaviour therapy*, *40*(2), 82-97.

6. Boutron, I., Moher, D., Altman, D. G., Schulz, K. F., Ravaud, P., & CONSORT Group*. (2008). Extending the CONSORT statement to randomized trials of nonpharmacologic treatment: explanation and elaboration. *Annals of internal medicine*, *148*(4), 295-309.
